# Machine Learning and Network Analyses Reveal Disease Subtypes of Pancreatic Cancer and their Molecular Characteristics

**DOI:** 10.1038/s41598-020-58290-2

**Published:** 2020-01-27

**Authors:** Musalula Sinkala, Nicola Mulder, Darren Martin

**Affiliations:** University of Cape Town, School of Health Sciences, Department of Integrative Biomedical Sciences, Computational Biology Division, Anzio Rd, Observatory, 7925 Cape Town, South Africa

**Keywords:** Cancer genetics, Cellular signalling networks, Data integration, Machine learning, Predictive medicine

## Abstract

Given that the biological processes governing the oncogenesis of pancreatic cancers could present useful therapeutic targets, there is a pressing need to molecularly distinguish between different clinically relevant pancreatic cancer subtypes. To address this challenge, we used targeted proteomics and other molecular data compiled by The Cancer Genome Atlas to reveal that pancreatic tumours can be broadly segregated into two distinct subtypes. Besides being associated with substantially different clinical outcomes, tumours belonging to each of these subtypes also display notable differences in diverse signalling pathways and biological processes. At the proteome level, we show that tumours belonging to the less severe subtype are characterised by aberrant mTOR signalling, whereas those belonging to the more severe subtype are characterised by disruptions in SMAD and cell cycle-related processes. We use machine learning algorithms to define sets of proteins, mRNAs, miRNAs and DNA methylation patterns that could serve as biomarkers to accurately differentiate between the two pancreatic cancer subtypes. Lastly, we confirm the biological relevance of the identified biomarkers by showing that these can be used together with pattern-recognition algorithms to accurately infer the drug sensitivity of pancreatic cancer cell lines. Our study shows that integrative profiling of multiple data types enables a biological and clinical representation of pancreatic cancer that is comprehensive enough to provide a foundation for future therapeutic strategies.

## Introduction

Pancreatic cancer is a heterogeneous disease that is characterised by poor clinical outcomes and few effective treatment options. Attempts to define a standard classification for tumours of the pancreas have been ongoing for decades^1–3^. In general, the approaches that are currently used for making both outcome predictions and treatment decisions are based on histological subtyping and clinical parameters such as the disease stage, metastasis, and the resectability of tumours^4,5^. Recently, however, the advent of molecular profiling has laid the foundation for quantitatively profiling tumours based on their genome-wide gene transcription profiles, protein expression profiles and/or mutational landscapes^6–9^. These profiling methods promise a more accurate and precise definition of tumour subtypes and better predictions of how particular tumour types will respond to different treatments.

Further, molecular data that is used to construct the molecular profiles of particular cancers have been used to identify the perturbances in the cellular regulatory networks that characterize these cancers: often revealing numerous potential drug targets within various signalling pathways. This molecular data together with the known molecular profiles of numerous well characterized cancer cell lines can even be leveraged using machine learning methods to predict the responses of particular patient tumour subtypes to different anticancer drugs^10,11^.

A crucial resource for the discovery of useful diagnostic biomarkers and potential anticancer drug targets are large-scale datasets comprising, among other data types, extensive genomic, transcriptomic and proteomic profiles of matched healthy and tumorous tissues. These datasets, which are compiled and maintained by The Cancer Genome Atlas (TCGA) and the International Cancer Genome Consortium (ICGC) are helping us uncover the molecular characteristics and signalling pathway perturbations that define specific cancer subtypes^12,13^.

Among the cancers that are well represented in these data collections is pancreatic cancer. Molecular profiling analyses of the pancreatic tumour datasets have identified both distinct pancreatic cancer subtypes, and mutations of the genes, KRAS, TP53, SMAD4 and CDKN2A as potential drivers of pancreatic cancer^14–18^. Although the biomarkers that differentiate between different pancreatic cancer subtypes could eventually inform treatment decisions, there are as yet no available subtype-specific treatment options for this type of cancer. There is, therefore, a pressing need to, firstly, find a set of biomarkers that can be used to accurately and sensitively diagnose pancreatic cancer subtypes and, secondly, to identify suitable targets for drug development among these biomarkers.

Definitions of disease subtypes is a perpetual process, with classifiers and cut-offs that differentiate between the subtypes, essentially needing to be continually re-defined and refined as more molecular data and better molecular profiling tools become available. As classification schemes for pancreatic cancers improve, it is expected that additional specific molecular correlates of patient survival, responses to anticancer drugs, and tumour aggressiveness will be uncovered. Armed with such knowledge, we could develop better prognostic and diagnostic methods, and select the best drugs to treat specific pancreatic cancer subtypes. Further, more subtype-specific molecular features could potentially enhance the accuracy with which machine learning methods could predict the drug response profiles of specific pancreatic tumours, thus leading to improved disease outcomes.

However, it remains technically difficult to effectively leverage the diverse and ever-increasing data relating to pancreatic tumours^19–21^. These difficulties include, but are not limited to, inconsistent classifications of patient tumours when the tumours are subtyped using different types of molecular data, and the efficient integration and analysis of different data types to yield consistent identifications of the causal disruptors of the molecular processes that underlie the observed differences between pancreatic cancer subtypes^19^. Ultimately, these difficulties undermine efforts to predict the responses of tumours to drugs: an endeavour involving comparisons between the relevant molecular features of a novel tumour with those of well-characterized tumour subtypes or tumour cell lines.

With these issues in mind, we attempted to identify clinically relevant subtypes of pancreatic cancer accounting for the full spectrum of molecular and clinical data available for pancreatic cancer tumours in the TCGA dataset. We address the problem of inconsistent tumour classifications that are obtained using different types of molecular data, by applying an integrative classification approach that considered all the available molecular data types. As expected, our analyses identified discrepancies between various classification schemes but ultimately supported the existence of two major pancreatic cancer subtypes. Besides uncovering the likely molecular causes of altered biological processes within the tumours of these two subtypes, we identified biomarker sets that can be used to accurately and sensitively classify novel pancreatic tumours. Further, in the face of multiple high-dimensional data types, we show that statistical models that capture the complexity of disease can aid in the identification of relevant drugs and drug targets that might offer substantial benefits for patients afflicted with tumours belonging to either of the pancreatic cancer subtypes.

## Results

### Subtypes of pancreatic cancer and their clinical characteristics

**Figure 1:**
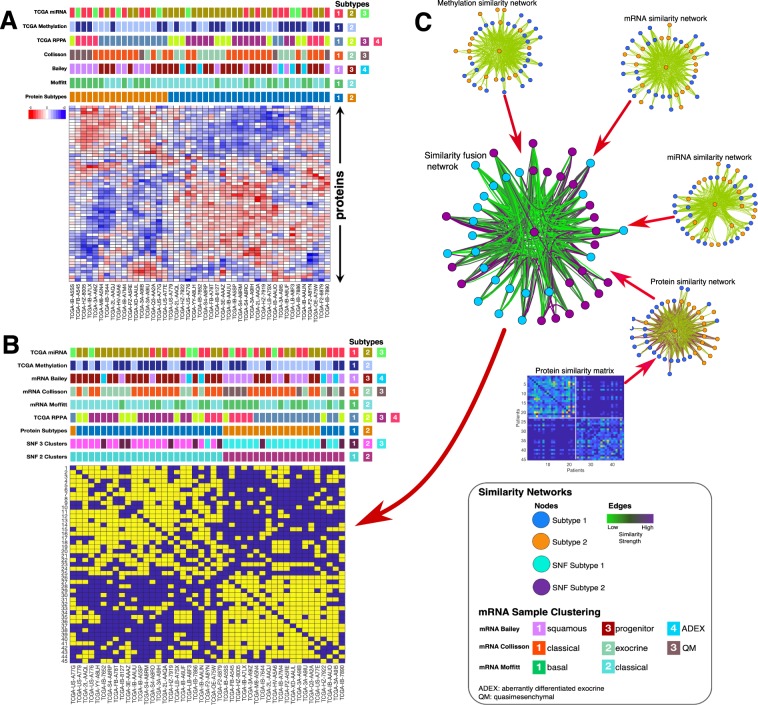
Classification of pancreatic cancer: (**A**) Comparison between the proteomics-based subtyping of pancreatic cancers using unsupervised hierarchical clustering, to other classification schemes from top to bottom: TCGA’s (Raphael *et al*., 2017) miRNA, RPPA, and DNA methylation; mRNA-based classification schemes using the gene biomarkers established by Collosson *et al*.; Bailey *et al*.; and Moffitt *et al*. (**B**) Illustrative example of SNF steps: similarity matrices are used to create patient networks from protein, mRNA, miRNA and DNA methylation data showing patient-to-patient similarities for the 45 pancreatic cancer patients. The network nodes represent patients. The colours of edges joining nodes indicate the degree of similarity between pairs of patients. The nodes of the fused network are coloured according to the subtypes to which the patient tumours were assigned using spectral clustering of the combined patient network. (**C**) Comparison between the SNF subtyping using spectral clustering to other classification schemes from top to bottom: TCGA’s^14^ miRNA, and DNA methylation classifications; mRNA-based classification schemes^8,48^; TCGA’s RPPA classification, our K-means clustering classification; our 3-cluster SNF classification; and our 2-clusters SNF classification.

We applied K-means clustering to the reverse phase protein array (RPPA) determined proteomics data of the 45 high-purity pancreatic cancer samples that are available in the TCGA database to identify two coherent clusters of patient tumours (Fig. S1A)^22^. Then, we compared this clustering of pancreatic cancer samples to other subtypes that are reported in the literature for various other molecular data types (DNA methylation status, protein expression levels and mRNA/ miRNA transcription levels) and established that the samples clustered differently depending on the specific molecular data type used (Fig. 1A).

To mitigate this problem, we applied a multi-platform integrative clustering method called similarity network fusion (SNF). SNF solves the disparate clustering problem by constructing similarity networks of samples for each available molecular data type and then efficiently fuses these into one network that represents clustering based on all the underlying data types (Fig. 1B)^19^. Using DNA methylation status, protein expression, mRNA transcription and miRNA data of the 45 high purity cancer tumour samples available in TCGA, we applied the SNF clustering method to identify two-cluster and three-cluster clustering solutions (Fig. 1C).

The pancreatic cancer subtypes in the two-cluster solution comprised 25 and 20 tumours, which we provisionally named as subtype-1 and subtype-2, respectively. Interestingly, the SNF clustering solutions were highly concordant with each of the clustering solutions obtained using individual molecular data types but were most similar to that obtained using the proteomics data (refer to Fig. 1C).

Next, we sought to understand whether the identified pancreatic cancer subtypes were associated with different clinical outcomes. Indeed, we found that the two groups of patients differed with respect to the overall percentages of individuals with progressive disease and the percentages of individuals who eventually died. Here we found that the patients with subtype-1 tumours were more likely to survive than those with subtype-2 tumours (75% vs 35% survival, respectively; Fig. S1C). We further observed a nearly 50% lower median disease-free survival (DFS) period for patients with subtype-2 tumours (DFS = 12.42 months) than for patients with subtype-2 tumours (DFS = 25.07 months; Fig. S1D). Likewise, the overall survival (OS) periods for the patients with subtype-2 tumours (OS = 16.05 months) were shorter than those with subtype-1 tumours (OS = 23.06 months; Fig. S1E). However, our analysis of OS and DFS periods using the Kaplan-Meier methods revealed no statistically significant difference between the pancreatic cancer subtypes; possibly due to the small sample size (Fig. S1D,E)^23^.

### Proteomics-based signalling pathway analyses distinguish disease subtypes

**Figure 2:**
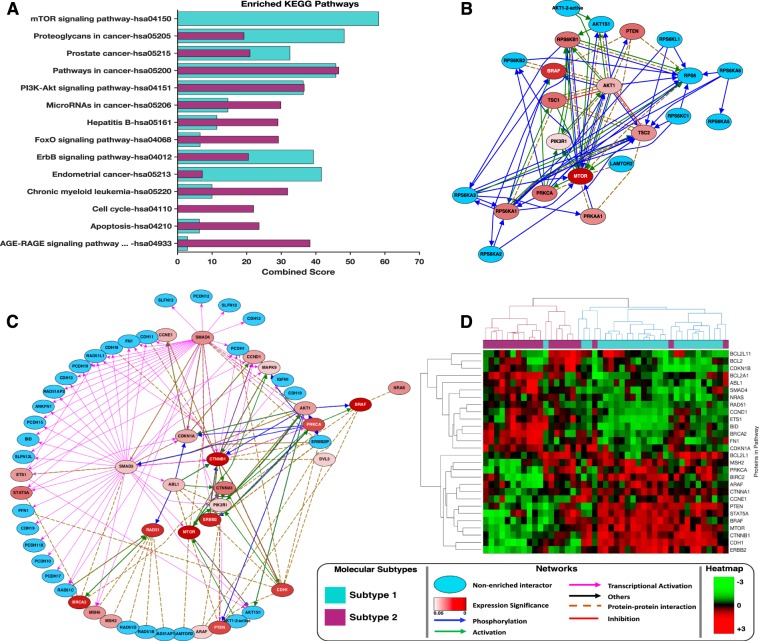
Pathway enrichment analyses: (**A**) KEGG pathways enrichment results of the most significantly altered pathways in tumours belonging to each of the inferred pancreatic cancer subtypes. Refer to Supplementary File 1 for the complete list of KEGG pathways enriched based on the proteomics data. (**B**) mTOR signalling pathways found to be uniquely altered in subtype-1 tumours. Blue nodes indicate proteins with expression levels that were either not significantly altered between the subtypes or were not measured by the TCGA. Red coloured nodes represent proteins with significantly altered expression levels with the degree of statistical significance being expressed as the negative logarithm of Benjamin-Hochberg adjusted p-values. The connectivity of network components was extracted from the KEA, ChEA, and UCSC super pathway databases. (**C**) KEGG cancer pathways found to be consistently altered in tumours belonging to both pancreatic cancer subtypes. (**D**) Clustergram of tumours using only the proteins that are members of the KEGG cancer pathways ontology.

For each disease subtype, we compared the enrichment of KEGG pathways and Gene Ontology (GO) biological process classifications of proteins that were upregulated within tumour belonging to each of the subtypes using Enrichr^24^. We found that whereas certain pathways were differentially altered between tumours belonging to different subtypes, other pathways were consistently altered (albeit to different extents in some cases) in the tumours of both subtypes (Fig. 2A, also see Supplementary File 1).

The mTOR signalling pathway was altered in subtype-1 tumours but not in subtype-2 tumours (combined score = 85, hypergeometric test; p = 2.1 × 10^−19^). Within the mTOR pathway of subtype-1 tumours, we found increased expression of well-documented oncoproteins including MTOR and BRAF: both of which have previously been linked to pancreatic carcinogenesis (Fig. 2B)^25–27^.

**Figure 3:**
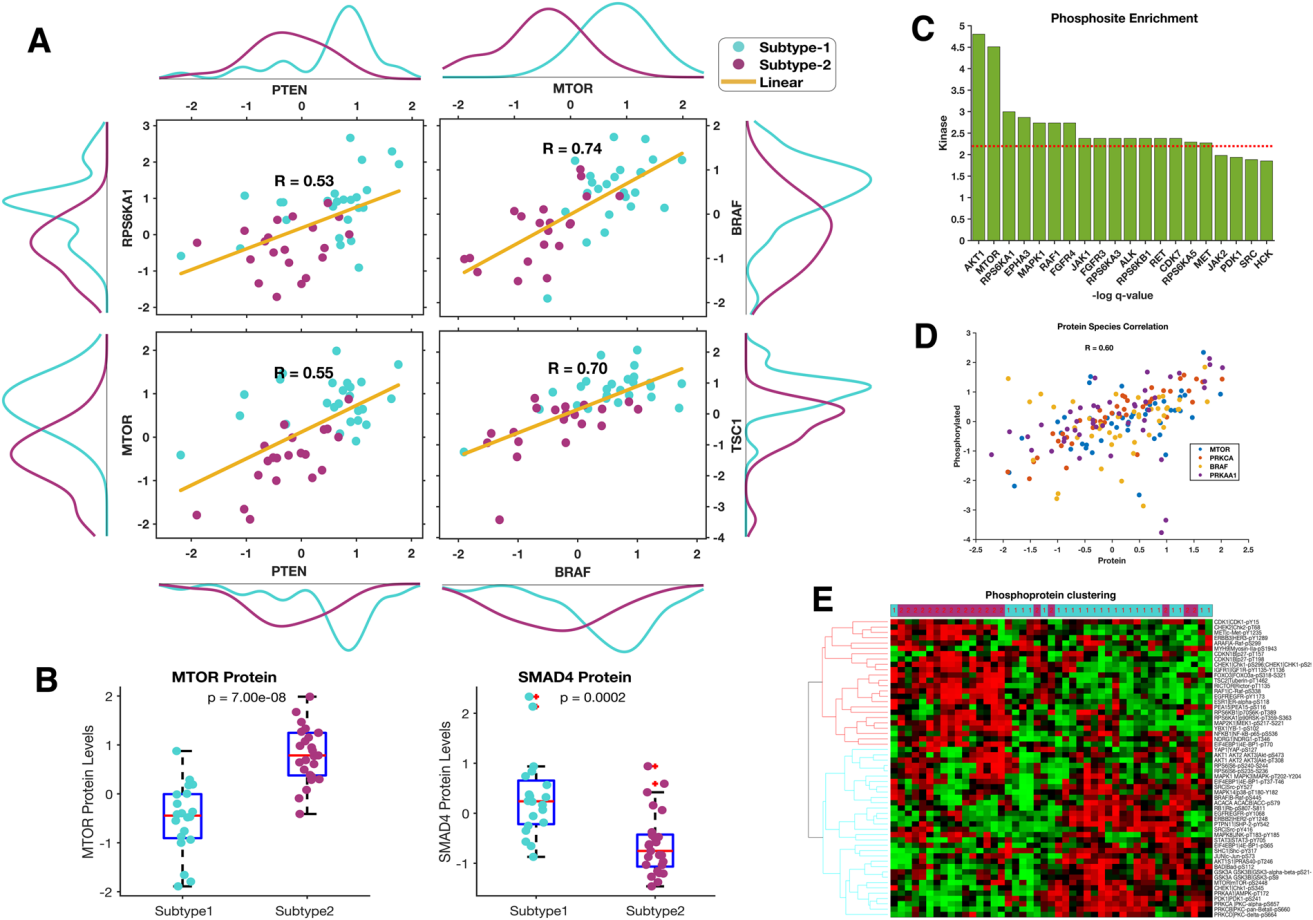
(**A**) Pearson’s correlation values of some proteins involved in mTOR signalling. The plot shows relatively higher expression levels of these proteins in subtype-1 tumours compared to subtype-2 tumours. (**B**) Boxplots show mTOR and SMAD4 protein expression biomarker of the SNF subtypes. (**C**) Enriched phosphosites identified by kinase enrichment analysis: the negative logarithm values of the Benjamin-Hochberg adjusted p-value are plotted on the y-axis while kinases are plotted along the x-axis. The red line represents the cut-off values at the 10% false discovery rate. (**D**) Correlation between the phosphorylated and de-phosphorylated proteins species for proteins involved in the mTOR signalling pathway. (**E**) Unsupervised hierarchical clustergram of tumours phosphoproteins showing high concordance with the clustering obtained from all the proteins (de-phosphorylated and non-phosphorylated protein) profiled by the TCGA. The clustergram was produced using the Spearman correlation distance metric and the complete linkage.

Further, we found that proteins that are involved in the KEGG Cancer Pathways were dysregulated in both the subtype-1 and subtype-2 tumours; these pathways encompass several known oncoproteins (such as RAD51, BRAC1, and ERBB2) and tumour suppressor proteins (such as PTEN and CDK2A1)^28–30^ (Fig. 2C). Despite the upregulation of the KEGG Cancer Pathways in tumours belonging to both subtypes, we found that the clustering of patients using only proteins within these cancer pathways was concordant with our subtype classification (Fig. 2D). Such a clustering pattern indicates that even when the same pathways are altered in both subtype-1 and subtype-2 tumours, the exact nature of the alterations within these pathways still differs between the two tumour subtypes. For example, whereas subtype-1 tumours exhibit hyperactivation of mTOR-associated signalling, subtype-2 tumours display increased activation of SMAD4-associated signalling. Also, we found that other proteins involved in mTOR signalling were both more strongly correlated and more highly expressed in subtype-1 tumours than they were in subtype-2 tumours, indicating the hyperactivation of this pathway requires the increased expression of most of the mTOR signalling proteins (Fig. 3A). Likewise, SMAD4 signalling pathway protein expression levels also differed significantly (p = 2 × 10^−4^) between these subtypes (Fig. 3B).

We further attempted to identify the kinases that likely phosphorylate substrates within the various signalling pathways of pancreatic tumour cells. Using kinase enrichment analysis (KEA), we found a subset of kinases that might drive pancreatic carcinogenesis, including, among others (Supplementary File 1), AKT1 (p = 8.2 × 10^−03^), MTOR (p = 0.011), and RPS6KA1 (p = 0.0499) (Fig. 3C)^31^. We observed a moderate positive correlation between proteins involved in mTOR signalling and their phosphorylated forms (Fig. 3D). Further, our results show that the protein phosphorylation pattern among the two pancreatic cancer subtypes is distinctive. Here, we found that in subtype-1 tumours various phosphoproteins that participant in mTOR signalling – such as MTOR-pS2448, GSKB-pS21-S9, PDK-pS241 and growth factor receptors EGFR-pY1068 and ERBB-pY1248 – all exhibited increased phosphorylation (Fig. 3E)^32,33^. These phosphoproteomics analyses support our initial findings (using dephosphorylated proteins) that subtype-1 tumours display increased mTOR signalling. Conversely, for subtype-2 tumours, we found elevated phosphorylation levels of proteins such as CDK1-pY15, p27-pT158 and p27-pT198 (Fig. 3E) which are involved in cell-cycle-associated processes^34^.

Overall, our findings suggest that for tumours of the two major pancreatic cancer subtypes, oncogenesis may be primarily driven by perturbation in either SMAD4 or mTOR signalling.

### Pancreatic cancer subtypes exhibit functional differences in mRNA levels and DNA methylation patterns

**Figure 4:**
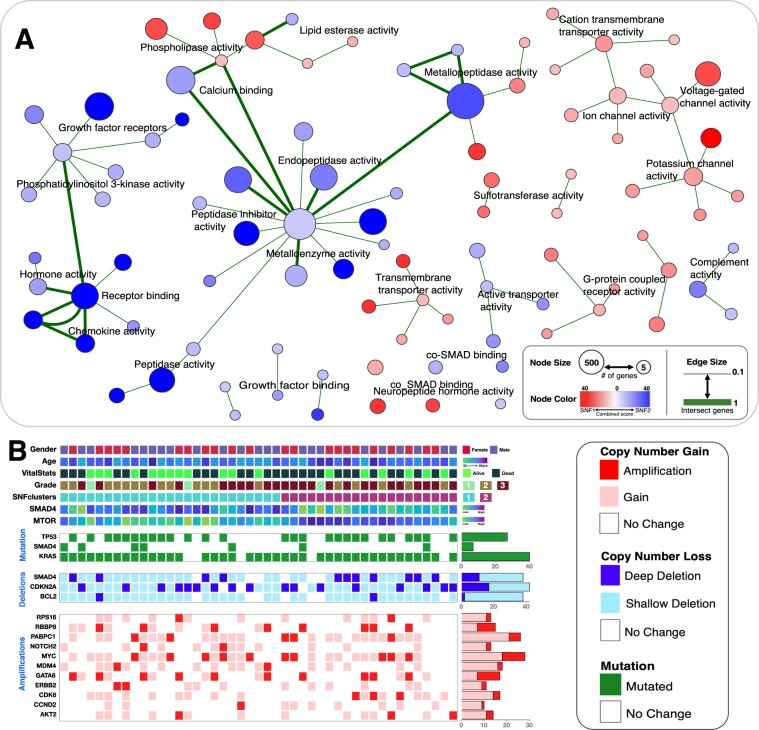
**(A)** Network of Gene Ontology (GO) molecular functions found enriched between the two pancreatic cancer subtypes. Enrichr was used to obtain enriched GO-terms that were visualised in Cytoscape (refer to the methods section). Each node represents a GO-term with similar nodes clustered together and connected by edges with the number of shared genes between the nodes being represented by the thickness of the edges. The size of each node denotes the gene set size of the represented GO-term. The colour of each node represents the magnitude of the combined enrichment score: red represent enrichment in subtype-1 tumours and blue represents enrichment in subtype-2 tumours. **(B)** The integrated plot of clinical and molecular features of 45 tumour samples ordered by their SNF clustering positions. From top to bottom panels indicate: patient gender; Age at which a condition or disease was first diagnosed; neoplasm histological grade; SNF subtype of tumour; SMAD4 protein expression level; mTOR protein expression level; significantly mutated genes: TP53, SMAD4 and KRAS gene mutations; SMAD4, CDKN2A and BCL2 gene deep deletion (dark blue) and shallow deletion (pale blue); gene amplification (red) and copy number gain (pink) of multiple genes.

We attempted to determine whether any GO molecular functions were enriched for among the overexpressed genes that differentiated the two pancreatic cancer subtypes. Specifically, we queried Enrichr using mRNA transcripts that were significantly upregulated across all of the tumours belonging to a particular subtype (see Supplementary File 2)^24^. We found that the over-transcribed genes in subtype-2 tumours were enriched for, among others, molecular functions associated with transmembrane transporter and G-protein coupled receptor activities (Fig. 4A, also see Supplementary File 1). Alternatively, the over-transcribed genes in subtype-1 tumours were enriched for, among others, molecular functions that are associated with phosphoinositide 3-kinase signalling, peptidase enzyme activity and growth factor receptors (Fig. 4A, also see Supplementary File 1).

We explored the enriched KEGG pathways that were differentially expressed between the two pancreatic cancer subtypes using lists of genes with methylation profiles and mRNA transcription levels that differed between the subtypes (see Supplementary File 2). Interestingly, we found that only subtype-1 tumours displayed enrichment for pancreatic secretions (Fig. S2A). These results corroborate both our previously noted enrichment in subtype-1 tumours of mRNAs involved in transmembrane transport, and published observations that the secretion of compounds from the pancreas and other organs is associated with increased transmembrane transporter activity^35^.

Similarly, for both enrichment analyses using differentially expressed mRNA and proteins, we found enrichment for components of the AGE-RAGE signalling pathway in subtype-2 tumours (Figs. 2A and S3A). The AGE-RAGE system promotes the development of various types of cancers, including those of the pancreas and prostate, through diminished apoptosis and increased cell viability^36,37^. Therefore, targeted inhibition of RAGE may serve as an effective treatment strategy against subtype-2 tumours.

In addition to these findings, the DNA methylation data revealed that while the methylation landscapes of subtype-1 and subtype-2 tumours were generally similar, the subtype-1 tumours had some additional genes displaying significantly increased DNA methylation (Supplementary File 2). We noted that these hypermethylated genes participate in various cellular pathways including focal adhesion, RAP1-signalling, and actin cytoskeleton regulation (Fig. S2B). Since these DNA methylation alterations are unique to subtype-1 tumours, they could be associated with reduced pancreatic tumour aggressiveness.

Unexpectedly, we observed no significant differences in mutation distributions and gene copy number alterations for the genes with transcription and translation profiles that differed between the two subtypes (Fig. 4B).

### Biomarker genes, proteins and miRNA sets that define the pancreatic cancer subtypes

Given that different types of molecular data yield different patterns of tumour clustering, we attempted to identify biomarker genes, proteins or miRNA sets that best differentiated between the two pancreatic cancer subtypes. It was anticipated that these sets of biomarker genes might allow for consistent classification of pancreatic cancer patients using machine learning methods applied to only one category of molecular data.

To extract relevant features for each category of molecular data, we applied the diagonal adaptation of neighbourhood component analysis (NCA) for classification with regularisation^38^. NCA learns feature weights for minimisation of an objective function that measures the average leave-one-out classification loss over the training data (Fig. S3A,B)^38^.

**Figure 5:**
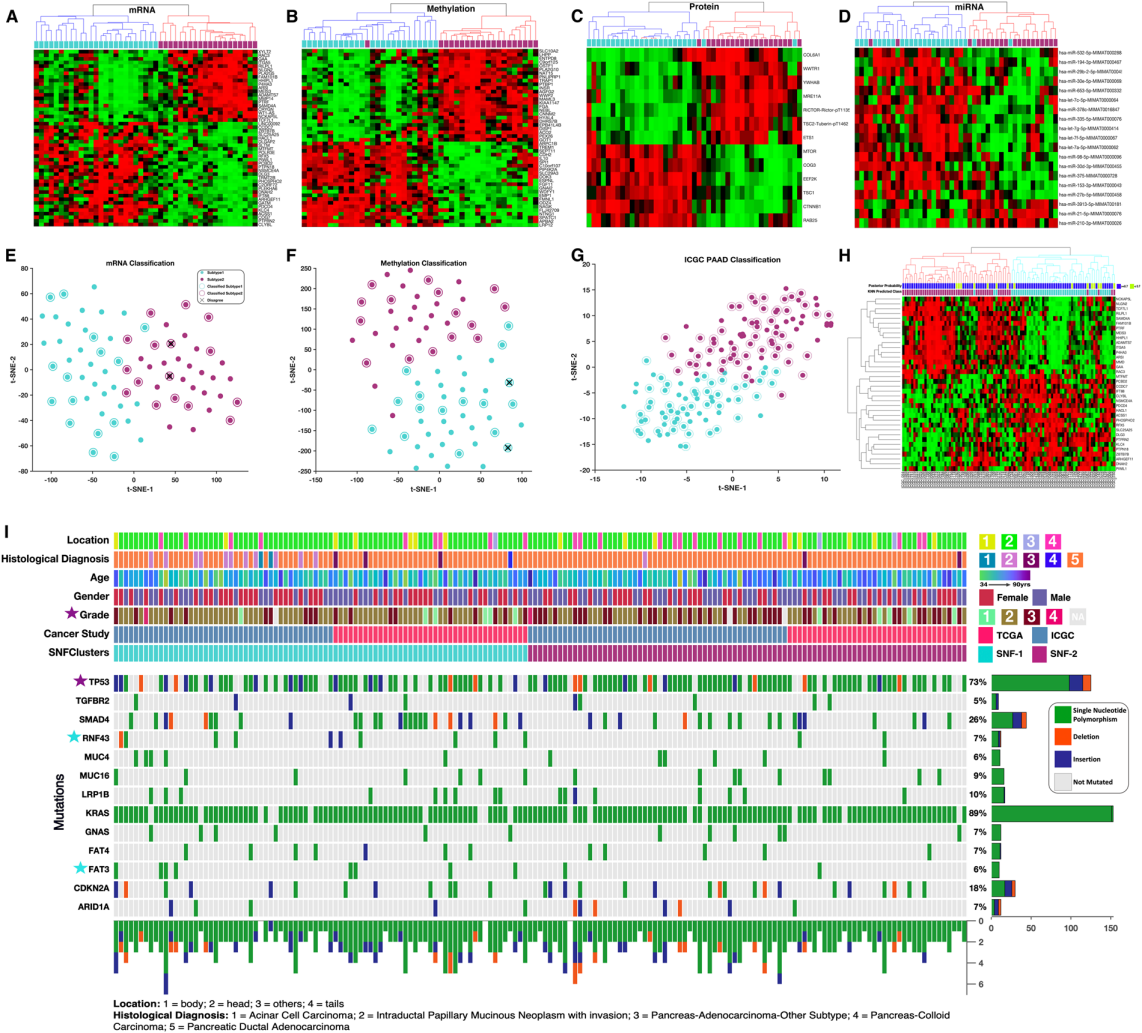
Clustered heatmap of tumours using the **(A)** mRNA biomarker gene set, **(B)** DNA methylation biomarker gene set, **(C)** protein biomarker set, and **(D)** miRNA biomarker set. All the heatmaps (In **A**–**D**) were produced using unsupervised hierarchical clustering with the cosine distance metric and complete linkage. The coloured bars on each clustergram shows the original subtype classification of each patient’s tumour found by applying SNF and spectral clustering to all molecular data sets. **(E)** Supervised classification of cancer patients using the mRNA biomarker set trained on the KNN-machine learning model. **(F)** Supervised classification of cancer patients using the DNA methylation biomarker set trained on an SVM-machine learning model. For both plots (**E**,**F**), t-SNE was used to visualise the tumour classes using the exact algorithm and squared Euclidean distance metric. Circled points represent newly classified TCGA pancreatic cancer patients, whereas un-circled points represent the original 45 tumour samples that were used to train the models. Crossed points represent disagreement between the mRNA-based model and the DNA methylation-based model. **(G)** Supervised classification of ICGC cancer patients using the mRNA-based KNN model trained on TCGA data. Circled points represent newly classified ICGC pancreatic cancer patients, whereas un-circled points represent the original 45 TCGA tumour samples that were used to train the model. **(H)** Unsupervised hierarchical clustering of the ICGC patients using the mRNA biomarker gene. The coloured bar on the clustergram shows the KNN model predicted class. (**I**) The integrated plot of clinical and molecular features for the TCGA and ICGC patient’s data, ordered by their integrative (SNF) clustering. From top to bottom panels indicate primary tumour location; neoplasm histological type; patient gender; age at diagnosis; neoplasm histological grade; cancer study; integrative tumour subtypes; non-silent gene mutations. The key to the number coding of tumour location and histological diagnosis is at the bottom.

Using NCA, we identified biomarker sets comprising 50 mRNAs, 49 methylated genes, 14 proteins, and 20 miRNAs. For these biomarker sets, we separately applied hierarchical clustering to each of the different molecular data categories to consistently and accurately reproduce the pancreatic cancer subtype classifications (Fig. 5A–D). Also, we individually applied supervised machine learning methods to the 50 mRNA, and the 49 methylated gene sets to classify tumours into subtype-1 and subtype-2 categories. For this, we used the K-nearest neighbour (KNN) algorithm for the mRNA expression data and the support vector machines (SVM) classifier (see methods section) for the DNA methylation data to achieve very accurate subtype classifications of the tumours (Fig. 5E,F)^39,40^. Specifically, we observed five-fold cross-validation classification accuracies of 99% for the mRNA-based KNN classifier and 98% for the DNA methylation-based SVM classifier, with an agreement of 97% (see methods sections).

Decreasing the number of biomarker genes needed to accurately classify tumours from new pancreatic cancer patients would improve the utility of these sets in a clinical diagnostic setting. To identify smaller biomarker gene sets, we used supervised machine learning methods (see methods section) to define a biomarker set of fewer than ten genes, miRNA or proteins that would minimise incorrect classifications (Fig. S3E). Also, we used these biomarker sets to consistently re-classify TCGA pancreatic cancer patients using hierarchical clustering (Fig. S3F–I). These results imply that smaller gene sets could potentially be useful in a clinical diagnostic setting.

To validate the performance of our 50-mRNA biomarker set, we downloaded pancreatic cancer data from the ICGC data portal^12^. Using the mRNA-based KNN classifier that was trained on TCGA data, we tested the reproducibility of the two-subtype classification scheme by classifying 96 ICGC pancreatic cancer patients into subtypes-1 and subtypes-2 (Fig. 5G). We also applied unsupervised hierarchical clustering to the mRNA biomarker set extracted from the ICGC RNAseq data to reproduce a two-subtype classification analogous to that obtained fusing the TCGA datasets (Fig. 5H). The grouping of ICGC patients yielded by the supervised “TCGA classifier” and the unsupervised “ICGC classifier” agreed on the classifications of 94% of the patients. We observed that 5% of patients with posterior subtype membership probabilities that were less than 0.7, were more likely to be among the discordant cases, accounting for five out of the seven discordant patients (Fig. 5H)^41^.

We examined mutational data for the genes that are frequently altered in pancreatic cancer together with the clinical features of subtype-1 and subtype-2 tumours from all of the patients represented in the TCGA and ICGC datasets (Fig. 5I). Here, we found no significant differences in the gene mutations between the tumour subtypes (see Supplement Table 2). Also, we observed that no genes were consistently altered in all of the tumours belonging to either of the subtypes. Similar to other studies, we discovered that some tumours lack mutations in any of the frequently mutated genes^42,43^. This diversity in the mutational landscape of pancreatic cancer tumours is likely to complicate the discovery of broadly applicable treatment regimens that target driver mutations^43^.

Concerning histological features of tumours that might be useful for differentiating between the subtypes, we observed that only subtype-1 tumours displayed evidence of intraductal papillary mucinous neoplasm, whereas only subtype-2 tumours were categorised by histological inspection as being pancreatic adenocarcinomas (Fig. 5I). Further, we found that subtype-1 tumours tended to be assigned a lower grade than subtype-2 tumours (χ^2^ = 10.3, p < 0.01).

### Subtyping pancreatic cancer cell lines and predicting drug responses

**Figure 6:**
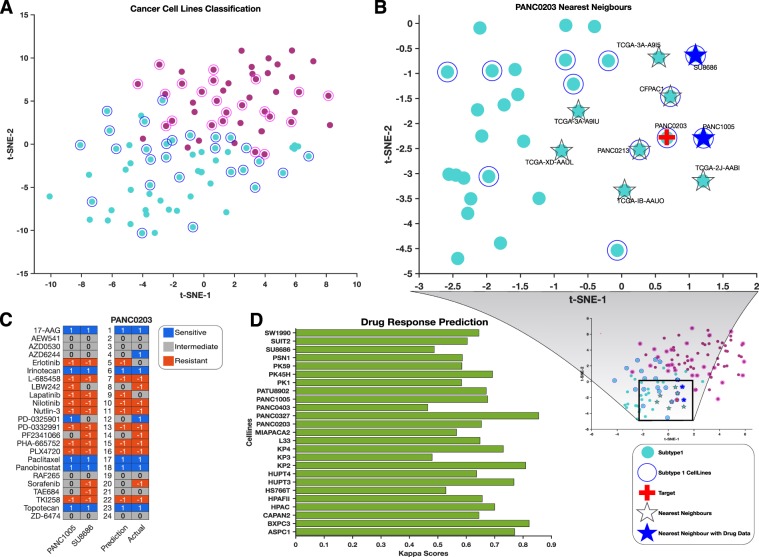
(**A**) Supervised classification of CCLE pancreatic cancer cell lines using the mRNA-based KNN-model trained on TCGA data. t-SNE was used to visualise the tumour classes using the exact algorithm and squared Euclidean distance metric. Circled points represent classified CCLE cell lines, whereas un-circled points represent the TCGA samples used to train the models. (**B**) The t-SNE plot represents the KNN search for the nearest neighbours of PANC0203 in the exhaustive searcher model. Refer to the legend at the right bottom of the figure for interpretation. (**C**) Drug response prediction: first two lanes represent the ranked drug responses to the 24 anticancer drugs of the PANC0203 nearest neighbours (PANC1005 and SU8686) for which such data is available. The last two lanes represent PANC0203’s predicted drug responses and its actual drug responses. (**D**) Kappa scores of all CCLE pancreatic cancer cell lines with drug data. The kappa score was calculated using the quadratic method by comparing the actual and predicted drug responses of cell lines to the 24 CCLE anticancer.

We obtained mRNA expression and drug response data for 45 pancreatic cancer cell lines from the Cancer Cell Line Encyclopaedia (CCLE)^10^. We attempted to subtype these cell lines using the biomarker gene set identified using the KNN classifier that we trained on the TCGA mRNA data (Fig. 6A). It is known that cell lines with similar transcription profiles are likely to exhibit similar responses to drug perturbations^10,44,45^. It follows, therefore, that the drug response profiles of cell lines should be predictable based on their gene expression profiles^10,45,46^.

We predicted the anti-cancer drug responses of the cell lines from the drug response profiles of the cell lines that are most similar (i.e., the nearest neighbours) to each “query” cell line as determined using an exhaustive KNN searcher model^47^. The Searcher model quantified and stored information concerning similarities between the transcription profiles of all the cell lines. Next, we retrieved the drug response profile of a “query” cell line and those of its nearest neighbours based on squared Euclidean distances from the Searcher model. To infer the drug response of the query cell line, we calculated the median drug response of the retrieved nearest neighbour cell lines to each of the 24 anticancer drugs that were profiled by the CCLE (Fig. 5B). For example, in Fig. 5B, the cell lines SU8686 and PANC1005 both have available drug response profiles in the CCLE database, and both are the nearest neighbours of the cell line, PANC0203. Therefore, we used the mean drug responses of SU8686 and PANC1005 to predict the drug responses of PANC0203 (see methods section) (Fig. 5C).

After predicting the drug responses of all the pancreatic cancer cell lines that also had observed drug response data, we compared the predictions to the observed drug responses. Our drug response predictions displayed substantial agreement with the actual drug responses in that they yielded an average Kappa statistic of 0.67 (Fig. 5D).

## Discussion

We conducted a comprehensive analysis of clinically relevant patterns of mutation, methylation, transcription, protein expression, and miRNA synthesis within pancreatic cancer tumours. Several pancreatic cancer studies have previously highlighted the limitations of utilising a single molecular data type to accurately classify pancreatic cancers (Fig. 2A)^8,9,14,48^. Here, we attempted to resolve this issue by employing a multidimensional clustering method capable of simultaneously utilising protein expression, mRNA transcription, DNA methylation and miRNA synthesis data. We found that by integrating across all these molecular data types, pancreatic cancer tumours could be classified into two clinically distinct subtypes: which we have simply named subtype-1 and subtype-2.

We observed that subtype-1 tumours were characterised by alterations of the mTOR signalling pathway, and the expression levels of different mTOR pathway proteins were positively correlated to each other (Fig. 1B–D). This finding is consistent with previous studies based on analyses of mRNA transcription and mutation data which also observed alterations of the mTOR pathway in pancreatic cancers^49–51^. Further, it is well established that some pancreatic cancer subtypes respond well to drugs which inhibit the mTOR pathway^52,53^. Accordingly, we anticipate that subtype-1 tumours will likely be more responsive to such therapies than will subtype-2 tumours.

Interestingly, subtype-2 tumours display unique alterations to cell cycle pathways (Fig. 1F,G). This is consistent with the observation that subtype-2 tumours are clinically more aggressive than subtype-1 tumours in that an element of aggressiveness is the hyperactivation of the cell cycle processes that accelerate tumour growth^54–57^.

We noted that, in addition to differences in patterns of protein expression, the two pancreatic cancer subtypes differ with respect to patterns of protein phosphorylation, implying that the kinases that are involved in oncogenic transformation differ between the subtypes. Specifically, whereas subtype-1 tumours show upregulation of mTOR signalling associated kinases (among others, MTOR-pS2448, GSKB-pS21-S9, and PDK-pS241), subtype-2 tumours display upregulation of cell cycle associated kinases (among others, CDK1-pY15, p27-pT158, and p27-pT198; Fig. 3E). Most of these kinases represent credible targets for small molecule inhibitors that might prove useful for subtype-specific anticancer therapies. Such small molecule kinase inhibitors are currently either being tested in clinical trials or are already in use as cancer therapies^57–60^.

In addition to displaying alterations in the mTOR signalling pathway, subtype-1 tumours also display evidence of elevated ion channel (Fig. 4A,D) and secretion pathway activities: a phenotype that is likely associated with increased trans-membrane transport of cell products (Fig. S2A). Changes in the expression patterns of ion channel proteins are also found in breast and prostate cancers^61,62^. In pancreatic cancers, ion channel proteins likely play crucial roles in cellular processes that are integral to oncogeneses such as cellular proliferation, motility, tissue invasion, and the excretion of lactic acid produced as a consequence of anaerobic respiration^63,64^. It is plausible therefore that subtype-1 tumours may be responsive to anti-cancer treatments that target ion channels and membrane pump proteins^63^.

Subtype-2 tumours on the other hand display elevated peptidase activities (Fig. 4A,C). Peptidases regulate various proteins that play essential roles in regulatory signalling networks. As is presently the case for tumours of the kidney, peptidases may be useful as diagnostic and/or prognostic biomarkers of subtype-2 pancreatic cancers^65,66^.

We found no significant differences in the mutational landscape between the two pancreatic cancer subtypes, indicating that the accumulation of similar genetic mutations drives the formation of tumours belonging to both subtypes. Recently, the paradigm of oncogenesis has been expanded beyond the classical view that oncogenesis is entirely driven by the accumulation of genetic mutations^67,68^. This paradigm now includes the disruption of epigenetic regulatory mechanisms and variations in miRNA expression^68–73^. Unlike with mutations, we currently lack adequate conceptual knowledge and the analytical framework needed to identifying putative driver and passenger changes in epigenetic and miRNA based regulatory processes^73–76^.

Nevertheless, we observed several differences between subtype-1 and subtype-2 tumours with respect to epigenetic (DNA methylation profile) and miRNA signatures. These suggest that epigenetic and/or miRNA variations may be primarily drivers of the differences in the transcriptome and proteome profiles of subtype-1 and substype-2 tumours.

In line with other studies that have identified biomarkers to classify tumour subtypes, some of which have important treatment and prognostic implications, we identified biomarker mRNA, DNA methylation, protein or miRNA sets that could be used to accurately subtype pancreatic tumours^8,9,48,77^. We are optimistic that any of these four biomarker sets could be individually used to obtain accurate subtype classifications for new pancreatic tumours. Nevertheless, the utility of these four biomarkers sets for predicting clinical outcomes and guiding treatment strategies will need to be evaluated in future studies.

Encouragingly, we were able to demonstrate that, by focusing on just the transcription levels of the mRNA molecules that are represented in our mRNA biomarker set, we could accurately predict the drug responses of cancer cell lines based on the drug responses of other cancer cell lines with similar mRNA expression profiles. Although others have also been able to predict the drug responses of cell lines using similar machine learning approaches^10,11,45,46,78^, our approach is novel in that it utilizes tumour subtyping based on all available molecular data to mine for biomarkers that differentiate disease subtypes: biomarkers which are then used to inform our KNN exhaustive search model with respect to quantifying the similarity of cell lines. What this means is that our approach is capable of utilizing matched molecular data and drug responses from either cancer patients or cell lines to predict, with reasonable accuracy, the drug responses of tumours for which we have only information on the concentrations of the mRNAs, proteins or miRNAs that are included within the biomarker sets which we have identified. As with other machine learning based inference schemes, the accuracy of the predictions that are made should improve given additional matched molecular and drug response data^79^.

Altogether, our analyses have revealed the molecular underpinnings of, and potential treatment strategies for, two clinically distinct forms of pancreatic cancer. We are optimistic that an approach such as we have used, where multiple different molecular data types are leveraged to subtype and characterise particular tumour variants, could yield valuable insights into the management of other difficult to treat cancers such as those of the lungs and triple negative breast cancer.

## Methods

We analysed data from 185 of the pancreatic cancer patients who had contributed samples to the TCGA project^13^. Data on these patient samples within the TCGA included: reverse phase protein array-based proteomics data (RPPA; n = 45), whole exome sequencing data (n = 76), transcriptome data determined using RNAseq (n = 76); DNA copy number and mutation data (n = 76), miRNA data (n = 56), and comprehensive clinical data. For our analyses, we only considered the 76 “high purity” samples for which transcriptome and whole exome sequencing data was available. Out of these 76 samples only 45 have RPPA data. All data used in our analyses were obtained from cBioPortal (http://www.cbioportal.org)^80^.

### RPPA-based Classification of Pancreatic Cancer

K-means clustering of proteomic data was performed to identify subtypes of the 45 high purity TCGA pancreatic tumour datasets with available RPPA data^39^. To find the most informative number of clusters, K-means clustering was run over 500 iterations for cluster sizes (K values) of two, three, four, and five (i.e., K = 2 to 5). The average silhouette values for each value of K were compared, revealing that the two-cluster solution had the highest mean silhouette value and was therefore deemed to be the most coherent (Fig. S1B). To aid in visualizing the most informative features that differentiated between the two inferred tumour subtypes, the 112 proteins with the highest entropy values across samples were used to reproduce the two-cluster K-mean classification using semi-supervised hierarchical clustering (Fig. 1A)^81^. The clustering pattern thus obtained was visualised using a principal component analysis plot (Fig. S1A)^82^. The clustering of these 45 pancreatic cancer tumours based on protein, miRNA and DNA methylation data has been previously published by Raphael *et al*.^14^, and the results of these clustering analyses were extracted from the supplementary file of that publication.

### Integrative Subtyping of Pancreatic Cancer

Similarity Network Fusion (SNF) is a clustering method that considers information from multiple molecular profiles. It has previously been used to segregate tumours of various cancer types based on multiple different sources of molecular data^19^. Briefly, standard normalised protein, mRNA, miRNA, and DNA methylation data derived from the 45 high-purity samples were used to create patient similarity networks (Fig. 2B). Next, we ran SNF to fuse the similarity networks over 25 iterations, with hyperparameter settings of 24 and 0.7 for the number of neighbours and alpha value, respectively. Finally, spectral clustering with two specified as the best number of clusters (identified according to the eigengap) was applied to the unified similarity network to obtain the final tumour classification (Fig. 2C)^19^.

### Patient’s clinical characteristics of the pancreatic cancer subtypes

The Kaplan-Meier method was used to compare overall survival and the duration of progression-free survival of patients with tumours belonging to the different pancreatic cancer subtypes^23^.

### Pathways and kinase enrichment analyses

The differentially expressed proteins between the pancreatic cancer subtypes were identified using the Student *t*-test with unequal variance and with the Benjamin-Hochberg correction applied to p-values^83,84^. Further, we queried Enrichr with two lists of 60 and 30 proteins found to be upregulated in subtype-1 and subtype-2 tumours, respectively, to return enriched KEGG pathways for each subtype (see Supplement File 1)^24,85^. The enriched KEGG pathways were compared to identify pathways that are unique to each of the disease subtypes^86^. The proteins that participate in pathways that are uniquely altered in sybtype-1 or subtype-2 tumours were used to construct protein-protein interaction networks using known interactions from each of the following databases: the University of California Santa Cruz Super pathway (101,525 protein-protein interactions), the Kinase Enrichment Analysis (428 kinases and their 10,792 targets), and Chromatin Immunoprecipitation Enrichment Analysis 2016 (667 transcription factors and their 464,967 targets)^31,87,88^. We visualised the resulting networks in yEd (Fig. 1B,C). Lastly, Kinase Enrichment Analysis was used to computationally identify the kinases that are responsible for the observed phosphorylation patterns in pancreatic cancer^31^.

The moderated student *t*-test based on the negative binomial model was used to identify differentially expressed mRNAs and variations in DNA methylation patterns (see Supplementary File 2)^89,90^. Additionally, functional enrichment analyses were performed using lists of differentially expressed mRNA transcripts or altered DNA methylation patterns associated with each disease subtype. These were used to query Enrichr to return Gene Ontology (GO) molecular functions and KEGG pathways enriched for each disease subtype (Figs. 4A, S2A,B, Supplementary File 2). A custom MATLAB script was used to create an enrichment network based on the enriched GO-molecular function designations. This enrichment network was visualised in Cytoscape(Fig. 4A)^91^.

### Identification and evaluation of biomarker sets

We used various data mining and machine learning methods to identify biomarker sets of mRNAs, DNA methylation, miRNAs or proteins that individually and consistently best stratified the two pancreatic cancer subtypes. The diagonal adaption of neighbourhood component analysis (NCA) with regularisation method was used to select the most useful features for each molecular data type^38^. Briefly, NCA attached feature weights to each attribute where the feature weights are used to select the most important attributes for classification. For each molecular biomarker dataset identified using NCA, unsupervised hierarchical clustering was applied to the TCGA datasets to reproduce the two-subtype pancreatic cancer classification (Fig. 5A–D). To apply supervised machine learning methods that accurately predict the tumour subtypes while utilising only one molecular data type, 23 different machine learning classifiers were trained ranging from linear discriminate analysis, support vector machines, decision trees, logistic regression, ensemble trees, and K-nearest neighbour algorithms. Then, the best performing classifier for each molecular biomarker dataset was selected based on their 5-fold cross-validation accuracy and area under the receiver operating characteristic curve. The selected models were the cubic K-nearest neighbour for the mRNA biomarker set (98.7% accuracy), quadratic SVM for the DNA methylation biomarker set (97.8% accuracy), Ensemble bagged trees for the protein biomarker set (95.6%), and the course Gaussian SVM for the miRNA biomarker set (93.3% accuracy)^92^.

To improve the accuracy of these models, the optimal hyperparameters that minimise the five-fold cross-validation loss were obtained using Bayesian hyperparameter optimisation (Fig. S3C,D)^93–95^. This improved the overall classification accuracy of the models on the cross-validation set to 100% for the mRNA-based KNN model and 99% for the DNA methylation-based SVM model. The trained models were then used to classify 31 other high-purity pancreatic tumours from the TCGA (Figs. 4F and 5E). Supervised learning models based on the proteomic or miRNA biomarkers datasets were not trained because there were too few other high purity samples profiled by TCGA for these data types. Further, for each molecular data biomarker set, between three and ten features were selected based on the lowest cross-validation loss of the best performing algorithm (Fig. S3E). These features were then used to classify TCGA pancreatic cancer samples using unsupervised hierarchical clustering (Figs. S3F–I).

### Validating biomarker molecular datasets

To evaluate the performance of the biomarker mRNA on a different pancreatic cancer dataset, we downloaded pancreatic cancer data from the ICGC data portal^12^. From the initial 50 mRNA biomarker set identified using the TCGA dataset, only 45 had corresponding data in the ICGC mRNA dataset. Therefore, we extracted the 45 gene biomarker set from both the TCGA and ICGC data. The mRNA-based KNN model was then re-trained on the TCGA 45 mRNA biomarker set. Here, standard normalisation was applied as a pre-processing step both to avoid platform associated biases, and because it was previously performed on the data before SNF clustering. Thereafter, the TCGA mRNA-based KNN model was used to predict the subtype of tumours in the ICGC dataset using a standard normalised mRNA biomarker set that was extracted from the ICGC RNAseq data (Fig. 5G). Also, unsupervised hierarchical clustering was applied to the ICGC biomarker gene set (Fig. 5F). Finally, the mutational landscape and clinical characteristics of the two pancreatic cancer subtypes of both the ICGC and TCGA datasets were compared (Fig. 5I).

### Subtype classification of cell lines

mRNA expression data from 45 pancreatic cancer cell lines together with their response profiles to 24 anticancer drugs were downloaded from the Cancer Cell Line Encyclopaedia^10^. The 50-mRNA biomarker set was extracted from the mRNA expression dataset and standard normalised. Then, the normalised CCLE mRNA biomarker genes were to subtype the cell lines by running the mRNA transcript levels for these genes through the mRNA-based KNN-model trained on TCGA data. The predicted subtypes of the CCLE cell lines were visualised using t-distributed stochastic neighbour embedding (t-SNE) (Fig. 6A).

### Machine learning method to predict a cell line’s drug response

An exhaustive nearest neighbour searcher model was created using standard normalised mRNA biomarker sets of both the CCLE cell lines and TCGA tumours^96^. The exhaustive searcher model takes as input the training data (in this case the mRNA biomarkers), distance metrics, and parameter values of the distance metrics for an exhaustive nearest neighbour search and can then be used to identify the nearest neighbours to a particular patient tumour or cell line within a specified radius of the distance matric. Here, the nearest neighbours to a particular cell line suggest similarity at the molecular level based on mRNA, DNA methylation, protein and miRNA data encoded in the SNF subtyping. The ten nearest neighbouring cell lines or tumours were determined using a nearest neighbour search algorithm based on a squared Euclidean distance metric (see Fig. 5B for intuition). After that, the drug response activity areas of the nearest neighbour cell lines were z-normalized and categorised as sensitive (for z-scored activity areas > 0.8), intermediate (for z-scored activity areas between 0.8 and −0.8), or resistant (for z-scored activity areas < −0.8). A simple prediction model was employed where the median responses to a particular drug of the nearest neighbouring cell lines was used to infer a target cell line’s drug response (Fig. 6B,C). Following this the quadratic Cohen’s Kappa score was used to evaluate the goodness of fit between the predicted and the actual drug response profiles of the cell lines (Fig. 6D)^97^.

### Statistical analyses

All statistical analyses were performed in MATLAB 2018a except where stated otherwise. Fisher’s exact tests were used to assess associations between categorical variables. Wilcoxon rank sum tests or independent sample Student *t*-tests were used for continuous variables where appropriate. Statistical tests were considered significant at p < 0.05 for single comparisons, and for Benjamini-Hochberg adjusted p-values < 0.05 for multiple comparisons.

### Ethics approval

The University of Cape Town; Health Sciences Research Ethics Committee (HREC) IRB00001938 approved the protocol of this study.

## Supplementary information

Supplementary Information.

Supplementary File 1.

Supplementary File 2.

Supplementary File 3.
