# Supplementary material for: Machine Learning and Network Analyses Reveal Disease Subtypes of Pancreatic Cancer and their Molecular Characteristics
Source: Sci Rep. 2020 Jan 27;10:1212. doi: 10.1038/s41598-020-58290-2 (PMC6985164; doi:10.1038/s41598-020-58290-2)
Supplement: Supplementary file 1 — Supplementary Information. [file 41598_2020_58290_MOESM1_ESM.pdf]

Machine Learning and Network Analyses Reveal Disease Subtypes of Pancreatic Cancer and their Molecular Characteristics

Musalula Sinkala
University of Cape Town
School of Health Sciences
Department of Integrative Biomedical Sciences
Computational Biology Division
Anzio Rd, Observatory, 7925
Cape Town
South Africa
smsinks@icloud.com

Nicola Mulder
University of Cape Town
School of Health Sciences
Department of Integrative Biomedical Sciences
Computational Biology Division
Anzio Rd, Observatory, 7925
Cape Town
South Africa
nicola.mulder@uct.ac.za

Darren Martin
University of Cape Town
School of Health Sciences
Department of Integrative Biomedical Sciences
Computational Biology Division
Anzio Rd, Observatory, 7925
Cape Town

South Africa

darrenpatrickmartin@gmail.com

Corresponding author e-mail address

smsinks@icloud.com

Supplementary Information

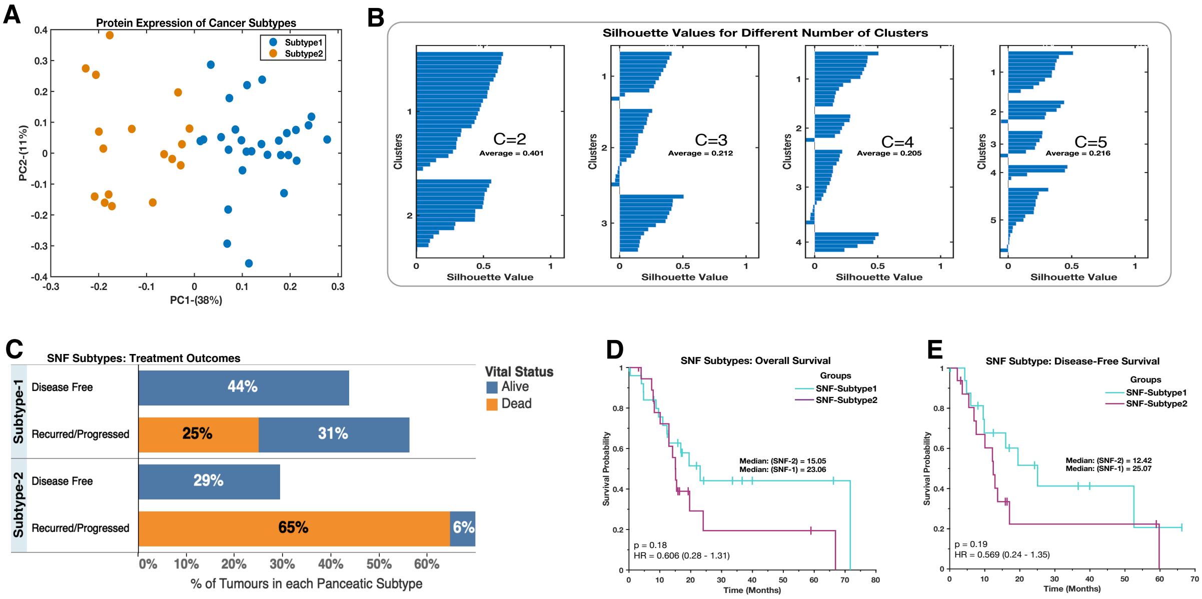

Figure S1: (A) Visualisation of the K-means clustering of the 45 high-purity pancreatic tumours. Principle component analysis was used to reduce the proteomic data dimensions, and the first two principal components plotted on the x-axis and y-axis, respectively. **(B)** Choosing the number of clusters: plots of Silhouette values for each value of K (number of clusters). **(C)** Vital statistics across proteomic disease subtypes for each treatment outcomes of either disease-free or progressive disease. Kaplan-Meier curves of the **(D)** period of disease-free survival and **(E)** the overall survival months of patients afflicted by integrative disease subtypes

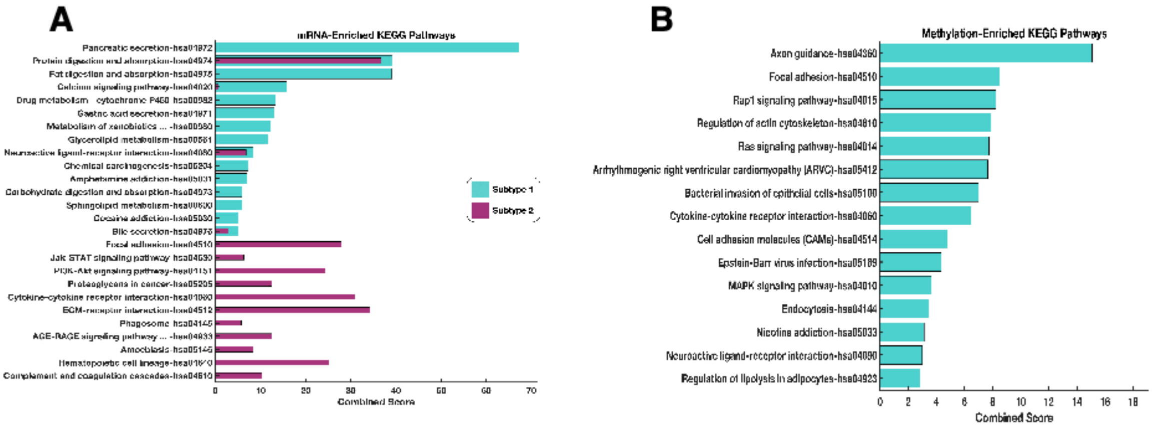

Figure S2: KEGG pathways: showing the top-ranked dysregulated KEGG pathways for each integrative disease subtype based on the **(A)** mRNA transcript levels, and **(B)** DNA methylation.

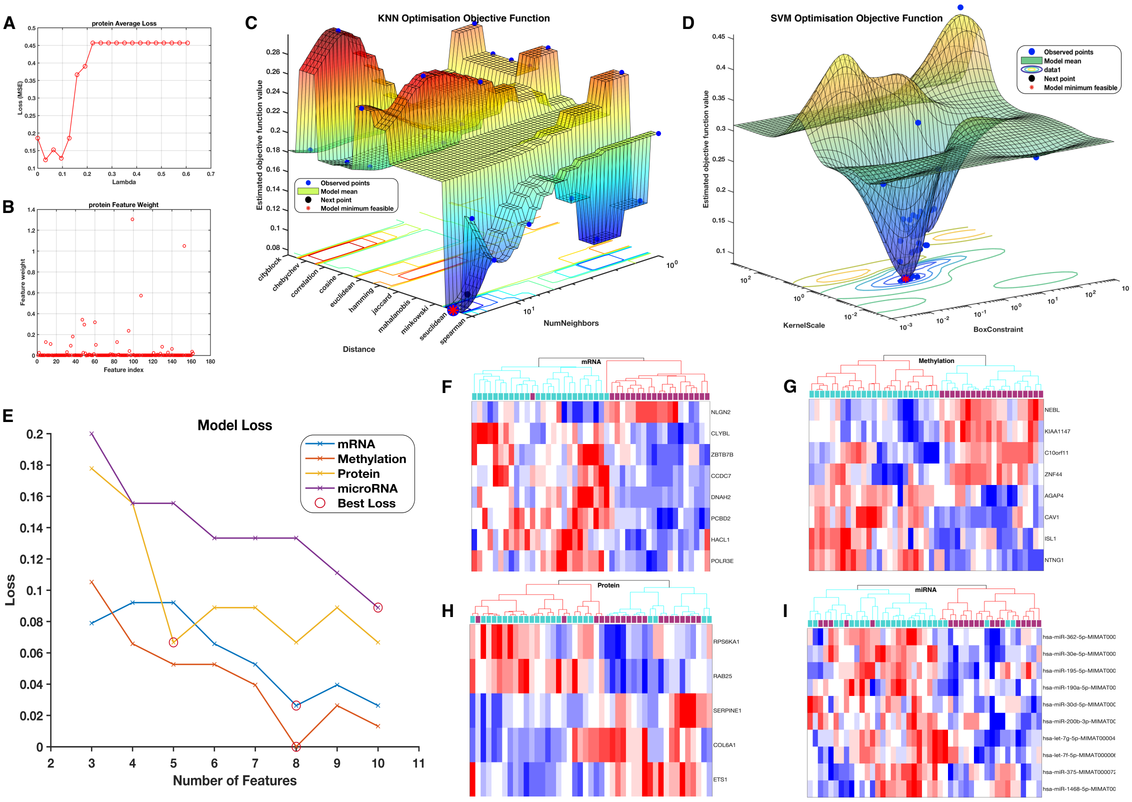

Figure S3: (A) Identifying the best regularisation value (lambda) for neighbourhood component analysis (NCA) for classification [1]: plot of average loss values vs the lambda values of classification. In this example, the best lambda value that corresponds to the minimum average loss for the protein features was 0.1203**. (B)** A plot of learned feature (protein) weights using NCA of the proteins in the RPPA data that would be used to classify patients into the two integrative subtypes. The weights of the irrelevant features are close to zero. **(C)** Plot showing the selected optimal machine learning hyperparameters using Bayesian optimisation for the mRNA-based KNN model [2,3]. On the x-axis are the number of neighbours, y-axis the distance metric and the z-axis average model loss. The optimal value is shown by the red star, i.e., one neighbour and the squared Euclidean distance. **(D)** Plot showing the selected optimal machine learning hyperparameters using Bayesian optimisation for the DNA methylation-based SVM model. On the x-axis is the box constraint, the y-axis is the SVM kernel scale and estimate average model loss on the z-axis. The optimal values are shown by the red star; box constraint of 0.46 and kernel scale of approximately 0.02. **(E)** The number of features plotted against the average loss

value of each supervised learning model for the Molecular data. The best in the top ten features that may be used to reproduce the integrative classification were selected based on the best loss (circled point). **(F)** Clustered heatmaps of the selected five mRNA transcripts, **(G)** four DNA methylation gene, **(I)** six proteins and **(J)** eight miRNAs: plots were produced using unsupervised hierarchical clustering with the correlation distance metric and the complete linkage.

Genetic Data	Biomarker Size	Best Algorithm	Accuracy	AUC
mRNA	50	Cubic KNN	98.4%	0.99
Protein	14	Ensemble Bagged Trees	95.1%	0.97
Methylation	49	Quadratic SVM	97.6%	0.99
microRNA	20	Course Gaussian SVM	93.3%	0.95

Table S1: Trained Supervised Learning Models

Machine learning models that were trained for each biomarker genes, proteins and miRNA. All models were training using 5-fold cross-validation, and the best performing models were selected based on the classification accuracy, and the curve area under the curve.

Gene	Chi Square	P-Value	Adjusted P-Value
ARID1A	0.333786103	0.563438152	0.843615793
CDKN2A	1.371503915	0.241553658	0.52336626
FAT3	3.8986332	0.048325408	0.209410101
FAT4	1.484768861	0.223029878	0.52336626
GNAS	0.00044137	0.983238629	0.983238629
KRAS	1.046475416	0.306320229	0.568880425
LRP1B	0.078952302	0.778722271	0.843615793
MUC16	0.25061223	0.616644317	0.843615793
MUC4	0.085064047	0.770548573	0.843615793
RNF43	5.837118963	0.0156915	0.101994749
SMAD4	3.219745336	0.072754948	0.236453581
TGFBR2	0.105266758	0.745598589	0.843615793
TP53	6.055228692	0.01386518	0.101994749

Table S2: Comparison of Gene Mutations Between Subtypes

Chi-square test results for the frequently altered genes between the two pancreatic cancer subtypes.

Supplementary File 1: Enrichment analyses of the KEGG pathways [4], Kinase Enrichment Analysis [5], and GO biological process [6] results for the subtypes of pancreatic cancer obtained using the differentially expresses proteins, mRNA transcripts, and DNA methylation.

Supplementary File 2: Differentially expressed mRNA transcripts, DNA methylation, proteins and miRNAs for the subtypes of pancreatic cancer.

Supplementary File 3: Biomarker sets of mRNA transcripts, DNA methylation, proteins and miRNAs that each may be used to classify pancreatic cancer patients into the integrative (SNF) subtypes.

References

[1] Yang W, Wang K, Zuo W. Neighborhood Component Feature Selection for High-Dimensional Data 2012. doi:10.4304/jcp.7.1.161-168.

[2] Snoek J, Larochelle H, Adams RP. Practical Bayesian Optimization of Machine Learning Algorithms. n.d.

[3] Gelbart MA, Snoek J, Adams RP. Bayesian Optimization with Unknown Constraints 2014.

[4] Kanehisa M, Furumichi M, Tanabe M, Sato Y, Morishima K. KEGG: new perspectives on genomes, pathways, diseases and drugs. Nucleic Acids Res 2017;45:D353–61. doi:10.1093/nar/gkw1092.

[5] Lachmann A, Ma'ayan A. KEA: kinase enrichment analysis. Bioinformatics 2009;25:684–6. doi:10.1093/bioinformatics/btp026.

[6] Gene Ontology Consortium: going forward. Nucleic Acids Res 2015;43:D1049–56. doi:10.1093/nar/gku1179.
